# Findings and recommendations from a root cause analysis of a clinical trial randomization error

**DOI:** 10.1017/cts.2026.10749

**Published:** 2026-05-15

**Authors:** Muayad Hamidi, Omar Abbaas, Pavan Bhatraju, Subrata Debnath, Jonathan Gelfond, Joel E. Michalek, Karen Nijland, Jeannette Watterson, Melanie Zuniga Rapp, Jason Bates, Susanne Schmidt, Kumar Sharma, Laura Aubree Shay, Hung-da Wan, Meredith Zozus

**Affiliations:** 1 Joe R. and Teressa Lozano Long School of Medicine, University of Texas at San Antonio Health Science Center (UT Health San Antonio), San Antonio, TX, USA; 2 Center for Advanced Manufacturing and Lean Systems, University of Texas at San Antonio (UTSA), San Antonio, TX, USA; 3 Department of Medicine, University of Washington (UW), Seattle, WA, USA; 4 School of Public Health, University of Texas Health Science Center at Houston (UT Health Houston), Houston, TX, USA

**Keywords:** Clinical trial, methods, procedures, guideline adherence, quality

## Abstract

**Introduction:**

Participants in NIH-funded multicenter clinical trial received the compound opposite to the randomization intention. We report the problem, subsequent root cause analysis (RCA) and corrective and preventative action (CAPA).

**Methods:**

The RCA was independently facilitated to identify study-level causes of the problem. Results were reviewed by institutional research administration leaders to identify institution-level causes and formulate corresponding CAPA. Both culminated in consensus.

**Results:**

The resulting causal chain consisted of two physical causes, two study-level system causes, and two institution-level system causes for which two institution-level CAPAs were formulated by institutional leadership. The CAPAs established institutional procedures for independent verification of critical processes, their specification, and their planned control on high-risk studies.

**Conclusions:**

Lack of study-level procedural infrastructure jeopardized a multicenter study led by our institution. Lack of institutional infrastructure supporting identification of critical study processes, risks to them, and appropriate controls enabled the problem to occur. Studies conducted in settings lacking institutional or study-level procedural infrastructure are susceptible to similar problems. Adding the needed institutional processes to support identification of critical study processes, risks to them, and appropriate controls required reprioritizing existing resources.

## Introduction

Clinical trials are complex endeavors [1]. Reports over the last two decades document that clinical trial complexity continues to increase [2–8]. It is well established in systems theory that complexity increases error [9–11]. Institutional processes for reporting and analyzing serious errors and incidents are expected practice in quality management standards such as the International Organization for Standardization [12] 9000 family of Quality Management System (QMS) standards [13] However, there are few[ 14–20] published reports of errors or problems in the conduct of clinical trials in the biomedical literature.

Based on experience and reports quantifying incident rates [3, 19, 21], errors and problems in clinical trial operations are common. While not necessarily all because of error, a large clinical trial Contract Research Organizations (CROs) reports that, “the majority of Phase I, II and III studies encounter at least one substantial amendment” [21]. The situation is no different for trials conducted by Academic Medical Centers (AMCs) [22]. As such, most clinical trials undertake surveillance, identification, and logging of protocol deviations, and all undertake reporting unanticipated problems involving risk to subjects or others (UPIRSOs). Most institutions conducting industry-funded, multicenter clinical trials for marketing authorization conduct a formal assessment of whether corrective action should be taken on the study and whether a change in the institution’s policy or procedure is needed when a consequential problem occurs. With close management, many problems are detected and corrected before adversely impacting studies or participants. Some errors and problems, however, escape detection prior to having an adverse impact on a study or study participants. With so few reports in the literature, other than the FDA inspections dashboard [23], there are no systematic and comprehensive compendia of common types of errors occurring in clinical trial conduct, much less causes or ways to prevent or mitigate them. The FDA dashboard lists lapses in regulatory compliance uncovered during an FDA inspection [23]. Thorough report and analysis of errors in clinical trial conduct are important because they may help others prevent or mitigate similar occurrences. We experienced an error in a key process on a clinical trial and are sharing the story, investigation, root cause analysis (RCA), and corrective and preventative action (CAPA).

### Background

Nicotinamide riboside (NR) in SARS-CoV-2 (COVID-19) Patients for Renal Protection (the NIRVANA trial, NCT04818216) was a randomized, double-blind, placebo controlled, multicenter clinical trial testing oral NR in hospitalized patients with COVID-19 infection and acute kidney injury. NIRVANA was conducted to determine the effect of NR on whole blood nicotinamide adenine dinucleotide (NAD+) levels and to evaluate safety of NR use in the studied population. Because some patients were expected to be intubated, two formulations of the study drug and placebo were manufactured: orally administered capsules and a powder to be dissolved by unblinded research site pharmacists for subsequent administration via nasogastric tube. Two of the three planned investigational sites were enrolling patients when an error was discovered.

Approximately six months into the study an astute unblinded, project officer noticed that draft tables for an upcoming Data Safety Monitoring Board (DSMB) meeting unexpectedly showed high NAD+ levels in patients in the placebo group and the reverse in the active group at one site. The study drug is a precursor to NAD+. Thus, elevated NAD+ levels were expected in patients on the active compound but not in the placebo group. After investigation, it was confirmed that the study patients receiving the capsule formulation of the study drug received the version opposite of that indicated on the randomization list. In other words, patients randomly assigned to active capsules received the placebo and vice versa. While study patients receiving the capsule formulation received the opposite of the randomization intention study patients receiving the solution form received the intended compound. The solution form was dispensed accurately because the site pharmacists were unblinded and knew whether they were dissolving the active compound or the placebo for administration. Complicating matters, at one site the capsule was primarily used, and at the other site, the solution was primarily used which made it appear as though the error was limited to one site. However, one patient at the site primarily administering the solution form was extubated and received both forms of the investigational product, that is, the active version of one form and the placebo version of the other.

The Investigational Product (IP) procurement, packaging, provisioning, and dispensing processes were retraced by an independent statistician to investigate the problem. Copies of communication, IP labels, packing slips, site IP accountability logs, the randomization list, and the randomization key were reviewed and matched. The independent statistician found that during study start-up, the unblinded statistician sent the randomization list to the site pharmacists in email and verbally confirmed with them that A was active and B was placebo. However, the manufacturer packing slip (not seen by the study statistician) listed A as placebo and B as active. The study statistician was not in communication with the manufacturer. The study statistician generated the randomization key with A as active and B as placebo. The study did not employ unblinded monitoring or unblinded randomization verification. Apart from the NAD+ levels to which the sites and study team were blinded, there was no way that the error could have been caught during the study.

**Figure 1.**
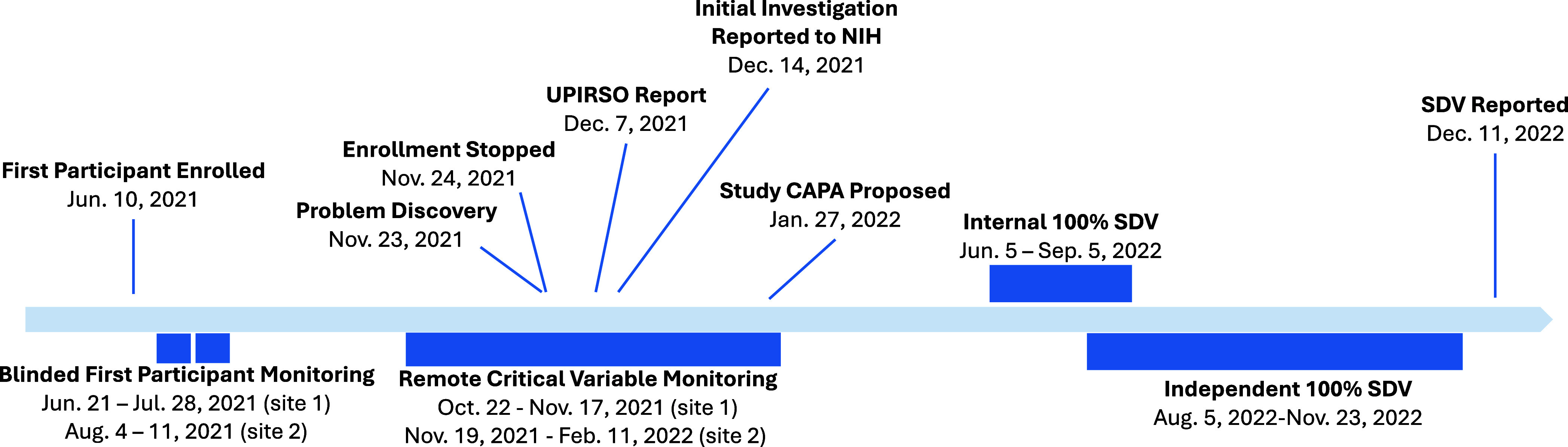
Figure 1 long description. Discovery and remediation timeline.

When the error was discovered, 28 participants had been enrolled at two of the three sites. Enrollment into the study was immediately stopped (Figure 1). A remediation plan was put in place and carried out, including changing the randomization key to match the IP labeling, updating the manual of operations, training site pharmacists on the incident and remediation, and independent verification of each patient’s study drug administration against the IP shipping information to confirm the actual administration for every patient. The funder subsequently required verification of all study data against the source documents at sites by independent monitors (Figure 1). The remediation actions and report were presented to the Data Safety and Monitoring Board (DSMB). The funder and study investigators ultimately agreed to end the study and perform analysis of the interpretable data.

Our objective here is to report the error, RCA, and CAPAs in hopes that such a report will help others prevent or mitigate similar occurrences.

## Materials and methods

The trial team, DSMB, and research funder agreed to involve our institutional Clinical Translational Science Award (CTSA) continuous quality improvement and evaluation team to conduct and disseminate an independently facilitated RCA of the error in the NIRVANA randomization process. Our CTSA process improvement team included two faculty with expertise in evaluation (SS, AS) and two systems engineering faculty at our institution (HDW, OA). All four faculty were independent from the seven study team members (MH, JM, SD, KS, PB, KN, and a site coordinator at Seattle no longer with the institution). Study participants were enrolled during hospitalization; clinical processes were used for investigational product (IP) dispensing and administration. The clinical pharmacists at each site delivered the IP (capsule or solution) to the nurse caring for the patient and nurses administered the drug to study participants. Beyond training on study IP dispensing, the sites’ pharmacists did not routinely meet with the trial team other than through routine clinical processes. Two institutional leaders (two authors, JG and MNZ) were involved in the remediation; the latter was additionally involved with the trial through oversight of study data management.

RCA is defined by the American Society for Quality (ASQ) as, “Root cause analysis is a process of drilling down to find causes of the problem so corrective action can be taken” [24]. The origins of RCA lie in attempts to prevent reoccurrence of American industrial accidents in the early 1900’s [25]. The principles of searching beyond symptoms to identify and correct the cause in underlying processes were honed and further developed in the USA and Japan by fathers of quality management such as Walter Shewhart, W. Edwards Deming, Kaoru Ishikawa, and Sakichi Toyoda [25].

Today, various methods and tools are applied to undertake RCA [26]. Frameworks for RCA in healthcare such as the Centers for Medicare and Medicaid Services [27] Quality Assurance and Performance Improvement (QAPI) RCA guidance [27] and The Joint Commission (TJC) Framework for Root Cause Analysis and Corrective Actions [28] vary considerably from general lists of steps (CMS QAPI) to detailed context specific tools (TJC). The former was too general and described in a brief twelve page guidance, and the latter was created with details relevant to healthcare events and lacked coverage of detail relevant to clinical research. The RCA reported here followed ten-step framework published by the ASQ[24]. Wording for the initial ASQ framework step, *define the problem*, was carefully chosen to precisely describe the unsatisfactory state without attributing cause, that is, clinical trial participants received the compound opposite to the randomization intention. The second ASQ framework step, *understand the process*, was accomplished by two institutional leaders (JG, MNZ) reviewing trial documents and interviewing team members to retrace and reconstruct the sequence of events preceding the apparent randomization error and documenting the events in a timeline (Figure 1). The event reconstruction was subsequently circulated among the trial team members to ensure accuracy, and to the independent evaluators (SS, AS) and systems engineers (HDW and OA) as input to the RCA.

The method used for step three in the RCA framework, *identify possible causes*, heeded the caution articulated by Paradies (2010) by first facilitating trial team brainstorming sessions to create a list of all possible causes and afterward following each successively deeper [24, 29]. The Suppliers, Inputs, Process, Outputs and Customers (SIPOC) method [30, 31] and an Ishikawa diagram [31] supported broad brainstorming and process mapping with the trial team to bring out as many possible causes as could be identified. Each identified cause was further questioned to solicit potential deeper causes [29]. The SIPOC method, Ishikawa diagram (also referred to as fishbone diagram), and process mapping were used to ensure systematic exploration of all possible causes. After no further causes were identified, that is, saturation was reached, participants were asked to independently rate each potential cause along two dimensions: (1) the importance or impact of correcting the cause to preventing similar events, and (2) the anticipated difficulty of correcting the cause. In addition, participants were asked to provide suggestions for corrective or preventive measures. The scoring results were displayed in a Possible, Implement, Challenge and Kill (PICK) chart, also called an Ease Impact Matrix, Impact Effort Matrix, or quad chart, a two-dimensional categorization according to the importance of correcting a cause *versus* the difficulty to correct the cause [32]. In addition, identified causes were grouped based on relatedness. The causes identified in the trial team RCA were sorted into commonly accepted categories: problem – the identified defect or failure that was observed; physical causes of the problem – actions or situations that directly resulted in the observed problem; and deeper system-level causes – those rooted in the way that an organization operates, that is, the underlying processes [24]. Only system-level causes were considered root causes [24]. Though physical causes such as a random event or a bad actor can occur in the absence of a system-level cause, if the problem was a critical one, there was a system-level cause of failure of the risk-management process, that is, failure to identify the critical risk and put prevention or detection measures in place, or failure of the prevention or detection measures. This is deeply rooted in Deming’s philosophy of quality management in that the real problems lie in the underlying process not in the people participating in the process [33]. Other categories such as contributing factors, that is, things, situations, or events that increase probability of problem occurrence but were not in the causal chain, as well as failure to predict the problem and failure to detect the problem were also documented [24]. Member checking was performed by all trial team members and RCA participants’ review of the results. This step three work was led by the systems engineers and evaluators in five sessions that took place between October 16, 2024, and January 13, 2025. All trial team members were invited to attend these RCA sessions and to complete the survey. A subsequent trial team survey was used to rate the impact of each cause and the anticipated level of difficulty taking corrective action.

As a form of peer review, and because root causes with potential to impact studies conducted elsewhere in the institution were identified, institutional research operational leadership at the director level and one level above were presented the historical account and trial team RCA results and asked to consider possible institutional-level root causes and CAPA. The review with institutional research administration leadership took place over the summer of 2025.

## Results

Of the seven trial team members, one was no longer at their institution at the time of the RCA and could not be contacted. Responses from the remaining team members and two institutional leaders involved in the study remediation were obtained in one or more of the RCA sessions. Five responded to the survey rating the impact versus difficulty of correcting the identified causes.

**Figure 2.**
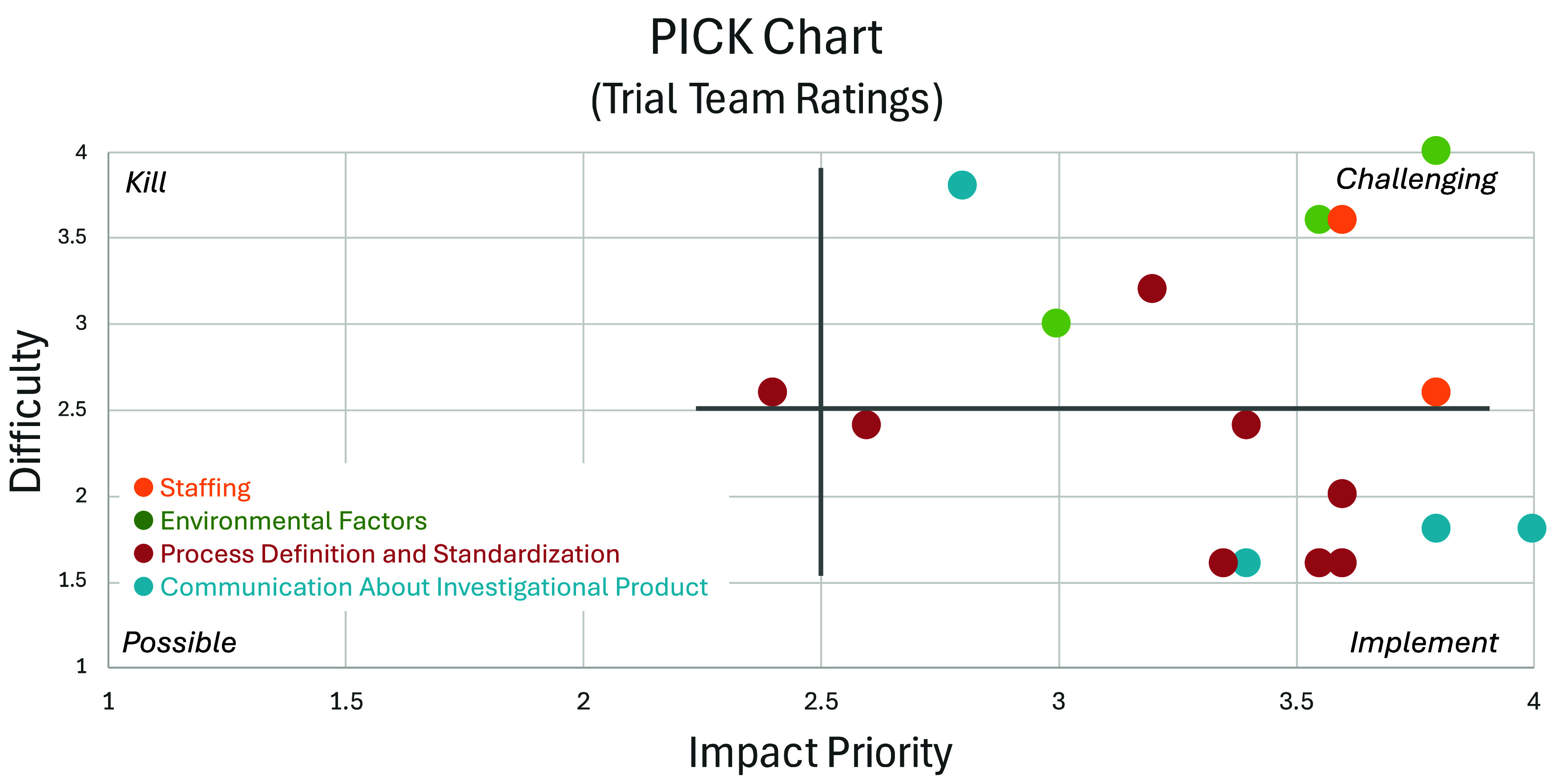
Figure 2 long description. PICK chart for identified causes. Three overlapping data points were jittered in Figure 2.

Seventeen causes were identified through the facilitated RCA sessions and categorized into four groups (Table 1, Figure 2). The three identified cause groups internal to the organization were: lack of process definition and standardization for key aspects of study operations, inadequate communication about the IP, and inadequate staffing. Two external causes were identified: time pressure secondary to the pandemic and study complexity. Limited funding, though subject to an external constraint, was not classified as external because organizations and study leaders in grant funded research choose the work they propose and take on for a given amount of funding.

**Table 1.** Identified causes Table 1 long description.

Cause	Classification	Priority (std. dev.)	Difficulty (std. dev.)
**Inadequate communication about investigational product**			
Investigational Product labels did not match the study randomization key (labels).	Physical cause	4.00 (0.00)	1.80 (1.30)
There was no communication between the IP manufacturer/packager and the study statistician.	Physical cause	3.80 (0.45)	1.80 (0.84)
Confirmation of IP label and randomization was not specified as part of the dispensing process.	System cause	3.40* (0.89)	1.60 (0.89)
Local site changes in pharmacist staff.	Contributing factor	2.80 (1.30)	3.80 (0.45)
**Inadequate staffing**			
Lack of a designated unblinded individual to check the randomization.	Detection	3.80 (0.45)	2.60 (1.34)
A professional Project Manager was not assigned to the study.	Contributing factor	3.60* (0.55)	3.6 (0.55)
**Inadequate process definition and standardization**			
Labels (for use by unblinded pharmacists) consisted of a generic single letter label rather than names such as “active” and “placebo.”	Contributing factor	3.60 (0.55)	1.60 (0.89)
IP packing slip was not cross-checked with randomization scheme.	Detection	3.60* (0.89)	1.60 (0.89)
Pharmacy manual and process lacked necessary detail and was not updated during the study.	Physical cause	3.60 (0.89)	2.00 (1.22)
Assignment of codes for active versus placebo was not covered in the pharmacy manual.	Contributing factor	3.40 (0.89)	1.60 (0.55)
No SOP for randomization that addressed investigational product package labeling.	System cause	3.40 (0.89)	2.40 (0.89)
Unclear process for developing the pharmacy manual.	System cause	3.20 (0.84)	3.20 (0.84)
Study data and activities, such as randomization, were conducted outside of REDCap.	Contributing factor	2.60 (0.89)	2.40 (0.89)
Study team members believed that REDCap email notifications may not be delivered successfully across institutional boundaries, such as due to variability in spam filtering.	Contributing factor	2.40 (1.14)	2.60 (0.89)
**Environmental factors**			
Limited funding led to limited resources.	Contributing factor	3.80 (0.45)	4.00 (0.00)
Time pressure due to the SARS-CoV-2 (COVID-19) pandemic	Contributing factor	3.60 (0.89)	3.60 (0.89)
Study complexity, for example, two different forms of the IP, critically ill study participants, and following patients discharged during treatment.	Contributing factor	3.00 (1.00)	3.00 (1.00)

Priority: 1 = low priority, 4 = high priority. Difficulty: 1 = easy to correct, 4=hard to correct.

* Overlapping points jittered in Figure 1.

Physical cause: actions or situations that directly resulted in the problem; system-level causes – underlying aspects of the way an organization operates, such as norms, culture, policy, and process, that allowed the physical causes to occur; contributing factor: things, situations, or events that increase probability of problem occurrence but which could not by themselves caused the problem to occur; detection: lack or failure of process controls; prediction: failure of risk management to identify implement process controls for a potential source of an impactful problem.

**Figure 3.**
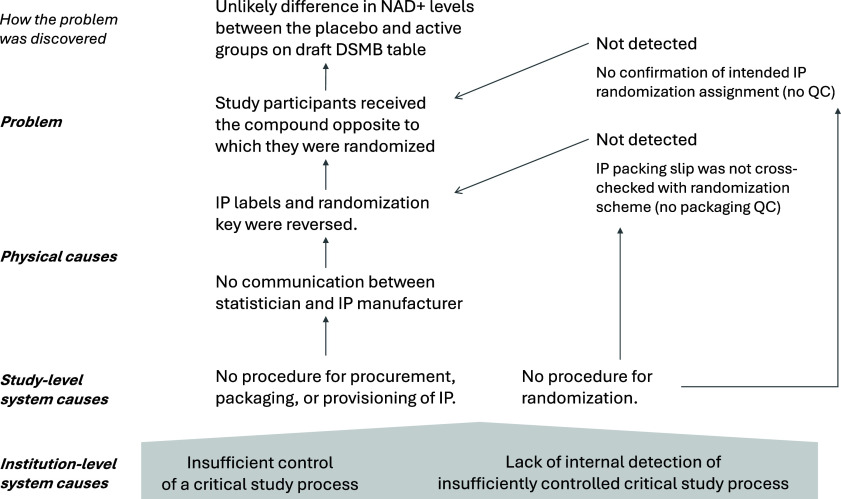
Figure 3 long description. Causal chain identified through the RCA.

The causal chain consisted of the problem, the physical causes, and the system causes. The problem, that study participants received the compound opposite of that to which they were randomized, had a single physical cause – the IP labels and randomization key were reversed. This physical cause was in turn caused by a second physical cause, the lack of communication between the study statistician and the IP manufacturer. Two study-level system causes were identified from the RCA results: (1) lack of a procedure for IP procurement, packaging, and provisioning, and (2) lack of a procedure for randomization (Figure 3). Since detection prior to study start could have prevented the problem, and detection during the study could have mitigated it, these causes (failed opportunities to detect the error) are shown to the right of the causal chain (Figure 3). The study-level system cause observed here could have been caught by the study team performing a mock non-blinded walk-through of the enrollment, randomization, and IP administration processes. This may have identified the mismatch between the randomization list code with the manufacturer’s codes. We have no institutional requirements for such a review and studies with limited time and resources are unlikely to conduct one on their own.

When asked to consider possible institutional-level root causes, leadership identified two institution-level system causes: (1) insufficient control of a critical study process, and (2) lack of internal detection of an insufficiently controlled critical study process. The former covers multiple study-level process definition and standardization causes identified by the trial team as well as staffing (Table 1) while the latter is distinct from the causes identified by the trial team (Figure 3). These two institution-level system causes were called out as needing remedy in the institution’s quality management approach for clinical studies.

### Institution-level cause 1

Insufficient control of a critical study process was deemed by leadership, through consensus, as the most impactful and root cause. The study processes for IP packaging and randomization were insufficient to ensure that study participants received the version of IP to which they were randomized. Lack of the norms commonly communicated through institutional and study-specific standardized procedures allowed inadequate flow of consequential information (the study statistician was not in communication with the drug manufacturer to specify allocation-relevant IP labeling information). Moreover, lack of a quality check step of confirming correct allocation allowed the problem to persist undetected.

How the error occurred, persisted, and eluded internal detection was explored. The IRB submission process at the time of the study included an investigator attestation of intent to comply with relevant laws, regulations, and other requirements which include Good Clinical Practice (GCP, ICH E6 R2 at the time). Written policy in effect now and at the time of the study, such as Policy RAQ 1.1.1 *PI Responsibilities*, and Policy RAQ 1.1.2 *Sponsor Investigator Responsibilities*, compile and summarize requirements relevant to sponsor-investigators. Relying on the attest approach alone requires investigators to understand and implement an identification of critical processes and design and implementation of procedures for them. Thus, at the time of the study and the RCA, designing and operationalizing a study capable of meeting all relevant requirements and ensuring that they were met was left to the study PI.

### Institution-level cause 2

Lack of institutional processes for early detection. Secondary to institution-level cause 1, institution-level cause 2 addresses the persistence of the problem on the study and lack of internal detection. At the time of the study, our university audit function did not cover coordinating center functions such as sufficiency of study procedures, their documentation, and their implementation. Thus, there was no independent readiness assessment of the central study team. A thorough readiness assessment may have detected a lack of adequate specification or control for a critical study process. An example of central readiness assessment is a table-top audit or protocol operational review prior to enrollment analogous to a site initiation visit but instead for the central coordinating function.

The two institution-level causes, (1) Insufficient control of a critical study process and (2) lack of internal detection of an insufficiently controlled critical study process, allowed the problem in question to occur. Specifically, the lack of an institutional mechanism to ensure that a critical study process was identified as critical, adequately specified, and adequately controlled, directly allowed the study to proceed without the study IP process being adequately written down and tested prior to the study, and without being checked during the study. These two institution-level causes lie far upstream from the specific study-level causes in that a whole host of inadequate study processes could go undetected because of causes 1 and 2. In other words, prior to the institutional CAPAs, any study team could fail to identify, specify, and control any critical process and the situation would, similarly to the case reported here, go undetected. This singular example demonstrates the importance of institutional processes and controls to ensure that study teams appropriately apply ICH E6 and E8 to thoughtfully identify Critical To Quality (CTQ) factors for each study, risks to the CTQ factors, and adequate prevention or controls.

### Proposed CAPA for cause 1

Upon considering the error and RCA, the convened research administration leaders recommended extending the institution’s role in prevention beyond reminding and holding investigators accountable for relevant laws, regulations, and requirements. The proposed CAPA includes the following three components. (i) Put an institutional process in place to identify greater than minimal risk, sponsor-investigator studies as part of the Just-In-Time (JIT) process or otherwise prior to accepting a study award. This is a time when the institution can assess and adjust alignment of the study scope and budget. (ii) At the time of award, and in coordination with the clinical department chair or dean, assign an experienced clinical trialist as a mentor for early career investigators or as peer independent eyes for a seasoned investigator, such that experienced investigator reviews sponsor-investigator responsibilities with the investigator and aids thorough identification of CTQ factors, supporting study processes and necessary controls. It was noted that a checklist of requirements created by the institution and based on the institutional policy would be ideal but would need to be developed. An existing risk management procedure with a tool for identification of CTQ factors was offered by an involved division. (iii) Add a corresponding prompt to document CTQ factors, risks, processes, and process controls in the QA/QC section of institutional clinical study protocol templates. This three-part recommendation supports investigators in identification, design, and control of the most important study processes before an omission becomes consequential and reinforces the necessity of study-specific procedures and controls for CTQ factors. The written study-specific procedures referenced in a study protocol would then be available to serve as a basis should the study be selected for audit. At the same time, this added support does not interfere with an investigator’s autonomy, authority or responsibility.

This CAPA is deliberately more general than the study IP procurement, packaging, provisioning, and randomization processes identified in the trial team RCA. This was deliberate. Additional oversight and support were more broadly deemed necessary for identification and management of CTQ factors. In other words, without such broad support in place, consequential CTQ factors and risks in areas other than the IP lifecycle could just as easily evade identification and preventative action. Thus, the institution-level causes and CAPAs are at a higher level of abstraction than. The specific process that failed on the NIRVANA study. Our cancer center had a process in place for the recommended early study feasibility assessment, others did not. Dedicating resources to instantiate this three-part recommendation in other areas across the institution necessitated reprioritization and reallocation of resources.

### CAPA for cause 2

The CAPA for the second institution-level system cause was to expand the scope of the institutional audit function to include internal or outsourced coordinating center functions for multicenter studies led by institutional sponsor-investigators. The decision to audit will be made according to the existing institutional risk stratification scheme, which is a scaled approach where high-risk studies are audited. Following the “at award” feasibility assessment, a pre-enrollment assessment will confirm that CTQ factors have been identified, and that study operations include specific procedures for their monitoring and control. Later during the study and where audit is justified by risk, compliance with stated study procedures can be assessed. Dedicating resources to instantiate this recommendation, as for the Cause 1 CAPA, necessitated reprioritization and reallocation of existing resources.

## Discussion

This case report provides an example of procedural infrastructure for clinical studies needed but lacking at AMCs. As a single case report, the likelihood of a similar incident cannot be generalized to other institutions. Neither can the causes nor the effectiveness of the CAPAs. However, a recent attempt to assess clinical trial management maturity, conducted at 23 AMCs (22 of which were CTSA institutions) reported an average self-rated maturity across the eleven axes of the rating tool of 2.54 out of 5.0 (SD: 0.50) [34]. On the newly developed Clinical Trial Ecosystem (CTE) maturity model used in the assessment, level 3.0 corresponds to having standardized procedures for clinical studies.

Community-wide efforts to build and improve research infrastructure in AMCs and for federally funded studies, while apparently incomplete, have achieved notable milestones. The Greenberg Report issued in 1967, addressed organization, review, and administration of cooperative studies [35]. Though, the report did not delve into study operations. In 1995 a special issue in the Controlled Clinical Trials journal (now called Clinical Trials) focused on study operations in academic studies with a collection of five review papers documenting current practice [36–40]. The first version of Good Clinical Practice (GCP), ICH E6, was published soon after in 1996 and provided the first international harmonization of clinical trial operational procedures. On January 1, 2017 (NOT-OD-16-148), GCP training became required for investigators and clinical trial staff involved in NIH-funded clinical trials. These are largely investigators and staff at AMCs. GCP (ICH E6) in addition to long-standing FDA regulation and guidance, consistently advocated and in increasingly required that written procedures be in place for clinical trial conduct.

The regulated industry answered the call by institutionalizing enterprise-wide standard operating procedures. However, at AMCs, PI authority, autonomy, and responsibility have been the norm. In academia it is the PI who justifies, designs, obtains the funding, and ultimately oversees study conduct. AMCs today continue to largely rely on PI-led study teams to determine and implement study procedures. Though institutional service centers usually standardize procedures for the services they provide, academic institutions remain reluctant to dictate how individual studies are carried out. AMCs are also reluctant to increase requirements on investigators due to already high investigator burden and limited resources. Ironically, lack of institutional procedural infrastructure places the full burden of procedure design, documentation, and implementation on individual investigators and relies on study budgets to cover the cost. By implementing some level of institutional standardization across clinical studies on which investigators could rely, some of this burden could be shifted to the institution, thereby limiting the investigator’s burden to those procedures that are truly study-specific.

The Inventory and Evaluation of Clinical Research Networks (IECRN) was initiated in 2004 as part of the NIH *Re-engineering the Clinical Research Enterprise* program [41]. It was the first national attempt to understand the level of infrastructure established in clinical research, though it focused on clinical research networks rather than institutions [41]. The Clinical and Translational Science Awards (CTSA) program, which funded its initial cohort in 2006, prompted and supported development of institutional research infrastructure and saw large successes such as widespread development of clinical data warehouses opening access to clinical data to researchers, almost ubiquitous adoption of REDCap [42], a large advance over then-current practice at AMCs for data collection and management in clinical studies, and institutional implementation of Clinical Trial Management Systems (CTMSs) and electronic IRB systems – all major improvements in clinical research infrastructure that would not likely have otherwise occurred. AMCs, however, remain idiosyncratic with respect to infrastructure to support the conduct of individual clinical studies. Today, this lies in stark contrast to expectations in the third revision of GCP, ICH E6(R3), which explicitly requires that clinical study processes that impact human safety or research results be identified, standardized, and controlled [43]. Clinical trial complexity and costs have continued to rise, while federal awards for research grants have remained flat. The scientific community, through consensus guidelines such as ICH E6, through regulation, and through award requirements has continued to increase expectations of rigor in study conduct. The situation forces inevitable choices for investigators. For example, adding professional project management in the budget or staff to draft extensive study-specific procedures means that fewer participants can be enrolled, fewer biological samples can be collected, or fewer parameters can be measured–in other words, less science. The balance between operational rigor and maximizing the science with limited funds is delicate. Consequences of too little of the former occur far into the future if at all and too little of the latter decrease likelihood of funding tipping the balance toward maximizing the science. This presents an ethical dilemma for investigators.

The study processes identified in the RCA as lacking definition and standardization: IP procurement, packaging, provisioning and dispensing, and randomization, would be considered by most to be critical study processes. At most AMCs today, these processes are designed, documented, and implemented by the trial teams working under the supervision of a multicenter clinical trial principal investigator rather than established by the institution as is done in industry-led clinical trials. In this setting, one way to obviate the root cause observed here would have been to initially perform a mock, non-blinded, protocol walk-through with review by the entire team and an institutional official. Such a review may have identified the mismatch between the randomization list code with the manufacturer’s codes.

National attempts to stabilize rising healthcare costs have continually increased the financial pressure on AMCs. Decreases in research indirect funds that often cover institutional research infrastructure, if implemented, will further increase the financial pressure on AMCs, evaporating resources that could have been reprioritized to develop institutional systems of policy and procedure for the conduct of clinical studies, leaving the burden on individual investigators and study budgets. The example presented here illustrates the procedural infrastructure gaps that existed at one institution. The demand for institutional research infrastructure has steadily grown to a level that many AMCs struggle to sustain. There is no indication that relief will materialize soon. Our solution to decreasing the risk of similar incidents, given the current reality at our, and other AMCs, is to bring expertise to investigators prior to study start as independent eyes to identify and close gaps between study plans and GCP requirements, and for high-risk studies to confirm sufficiency of plans through audit.

### Lessons learned from the RCA

While the causes in and of themselves are lessons learned, we had one additional methodological observation. RCAs remain underused, yet they are essential mechanisms for spreading risk awareness and driving improvements both within and across institutions. While our institution has multiple individuals trained in QI methods, the research QMS at our institution did not utilize or assign responsibility for RCA and CAPA outside an individual research division and the institutional UPIRSO process. This significantly increased the elapsed time between the incident and the formal RCA and lengthened the RCA itself. No matter how an institution’s research QMS is structured, we recommend an interdisciplinary incident response team to consult on immediate remediation, lead formal RCA for serious incidents, and ensure that institutional learning is sustained via institutional policy and procedure. This requires a process for incident recognition and reporting and an institutional culture of openness and transparency.

As a single case report, the causes and CAPA may not be directly transferrable to other research institutions or contexts. However, as an example of where lack of procedures including those for deciding and implementing process controls led to occurrence of a serious failure and subsequently failure to detect the problem, the case report provides evidence that a similar problem could happen in other institutions lacking procedures for the involved processes. The RCA methodology used is also likely to be valuable to other institutions. However, we note that the language is inconsistent across the many RCA frameworks. For example RCA is variously conceptualized as sometimes preceding, sometimes encompassing, and sometimes encompassed by the CAPA process. The CAPA processes further exists within broader quality management wisdom that consistently advocates for measurement and monitoring of process changes to ensure that the changes were and remain effective. However, problems of a severe but infrequent nature like the randomization error reported here are not amenable to this treatment. Lack of recurrence within one, three, or more years out from a correction cannot be taken as evidence of eradication. Thus, problems of an infrequent but severe nature require engineering prevention or multiple layers of defense into the underlying process, and one of the defenses may include a mechanism for early detection.

## Conclusions

Lack of procedural infrastructure jeopardized a multicenter study led by our institution. Lack of procedural infrastructure at AMCs for the conduct of clinical studies is common today. Institutions lacking procedural infrastructure are susceptible to problems like that reported here. Our CAPA represents a small increase in institutional process to support investigators in meeting GCP requirements for critical study processes. Sustaining resources to support investigators in meeting these reasonable expectations for identification, specification, and control of critical study processes necessitated reprioritization and reallocation of existing resources at our institution.

## Figure 1. Long description

The figure shows a horizontal timeline with significant events in the RCA process marked on it. The marked events include the following. The First Participant was Enrolled (FPE) in the NIRVANA study on June 10, 2021. Blinded clinical trial monitoring of the FPE at sites 1 and 2 took place from June 21 through July 28, 2021, and from August 4 through August 11, 2021 respectively. Remote critical variable monitoring at sites 1 and 2 took place from October 22 through November 17, 2021 and from November 19, 2021, through February 11, 2022, respectively. Thew apparent randomization problem was discovered on November 23, 2021. Enrollment on the NIRVANA trial was stopped on November 24, 2021. The Unanticipated Problem Involving Risks to Subjects or Others (called a UPIRSO) was filed with the University of Texas Health Science Center at San Antonio local Institutional Review Board (IRB) and the trial IRB of record on December 7, 2021. The findings from the initial investigation was reported to the NIH on December 14, 2021. The Corrective And Preventative Action Plan (CAPA) was proposed on January 27, 2022. Internal 100% Source Data Verification (SDV) was performed to calculate the database-to-source error rate from January 5 through September 5, 2022. Independent 100% SDV was performed as cases completed internal SDV from August 5 through November 23, 2022. The error rate results from the internal and external-independent SDV processes were reported on December 11, 2022.

Navigate back to Figure 1..

## Figure 2. Long description

Figure 2 is a PICK chart showing the average priority rating on the X axis rated from 1 to 4 where 1 is the lowest impact and 4 is highest, and average difficulty to correct rating on the Y axis, also rated from 1 to 4 where 1 is the least difficulty and 4 is the highest. The chart is divided into four quadrants labeled “P”, “I”, “C”, and “K”. P stands for Possible to correct where both impact and difficulty are the lowest. I stands for Implement where impact is highest and difficulty is lowest. In other words the causes in this quadrant should definitely be implemented. C stands for Challenge where both impact and difficulty are high. Causes in this quadrant will be harder to correct because the difficulty rating was high, but should be considered for correction because the priority was also high. K stands for Kill. These causes had a low priority rating and a high difficulty rating. On the PICK chart, 7 causes fall in the implement quadrant (high impact with low difficulty) with 4 of those causes categorized as “Inadequate Process Definition and Standardization”, and 3 categorized as “Inadequate Communication About Investigational Product”. Most of the remaining causes fall in the challenge quadrant of the PICK chart where both impact and difficulty are high.

Navigate back to Figure 2..

## Table 1. Long description

Table 1 consists of 4 columns and 17 rows (each row is a cause). The columns headers are cause, cause classification, priority, and difficulty. The causes were further categorized into 4 categories as follows: The first cause category, “Inadequate Communication About Investigational Product (IP)”, contained four causes. 1. IP labels did not match the randomization key (this one scored the highest priority: 4.0) 2. No communication between manufacturer and statistician 3. No confirmation of labeling during dispensing 4. Local site changes in pharmacist staff. The second cause category, “Inadequate Staffing”, contained two causes. 5. No designated unblinded person to verify randomization 6. No assigned project manager The third cause category, “Inadequate Process Definition and Standardization”, contained eight causes. 7. Poor labeling practices (generic labels instead of “active/placebo”) 8. No cross-checking of packaging with randomization scheme 9. Pharmacy manual of procedure was outdated and lacked necessary details 10. Assignment of codes “active” vs “placebo” was not covered in the MOP. 11. No SOP for randomization that addressed IP package labeling. 12. Unclear process for developing the pharmacy manual. 13. Study activities such as randomization were conducted outside REDCap. 14. REDCap notification emails were misconceived as undeliverable due to spam filtering. The fourth cause category, “Environmental Factors”, contained three causes. 15. Limited funding (this one scored the highest difficulty: 4.0) 16. Time pressure due to COVID-19 17. Study complexity (e.g., multiple IP forms, critically ill patients) The cause classification column labels each cause as physical cause, system-level cause, contributing factor, detection, or prediction. The cause classifications are defined as follows. Physical cause: actions or situations that directly resulted in the problem; System-level causes – underlying aspects of the way an organization operates, such as norms, culture, policy, and process, that allowed the physical causes to occur; Contributing factor: things, situations, or events that increase probability of problem occurrence but which could not by themselves caused the problem to occur. The Cause classification column labels each cause as Detection: lack or failure of process controls; Prediction: failure of risk management to identify implement process controls for a potential source of an impactful problem. The priority column contains the cause priority rating from 1 to 4 where 1=low priority and 4=high priority. The column also displays the standard deviation of the priorirt ratings for the cause in parentheses. The difficulty column contains the rating of the difficulty to correct the cause as rated by the study team from 1 to 4 where 1=easy to correct and 4=hard to correct. The column also displays the standard deviation of the difficulty ratings for the cause in parentheses. Key insights from the table include, (1) the highest priority issue was the mismatch between IP labels and randomization key; (2) the cause rated most difficult to fix was limited funding (difficulty = 4.0), and (3) the most types of causes were the contributing factors and system-level causes.

Navigate back to Table 1..

## Figure 3. Long description

Figure 3 shows the causal chain identified through the Root Cause Analysis (RCA). On the right of the diagram, the causal chain traces the sequence of events leading to the error, i.e., how the error happened. The deepest, most foundational causes are shown at the bottom. Physical causes are in. the middle, and the observed problem (the apparent randomization error) is at the top of the diagram. The failures in detection appear on the left of the diagram. While the detection failures are not causal, they could have interrupted the causal chain and prevented further occurrence. The detection failures are shown on the left to separate them from the causal chain. A step-by-step breakdown of the causal chain in the diagram: (1) Institution-level system causes (bottom) including Insufficient control and lack of internal detection of a critical study process. (2) Study-level system causes including no procedures for procurement, packaging, or provisioning of investigational product (IP), and no randomization procedure. The study lacked clear standard operating procedures (SOPs). (3) Physical causes including no communication between the statistician and IP manufacturer and IP labels and randomization key were reversed. (4) Problem (what went wrong): Participants received the wrong treatment (opposite of their assigned group). On the left side of the diagram, the missed opportunities to detect the problem, i.e., why the problems weren’t detected, include the following. Because there was no independent confirmation of randomization assignments, and no cross-checking of the IP packaging with the randomization scheme, the problem was not detected as early as it should have been. Even after the error occurred, no safeguards caught it. For reference, the diagram also lists how was the issue discovered (top of the diagram), i.e., the Project Officer noticed the unlikely difference in NAD+ levels between groups on draft tables for the Data Safety and Monitoring Board. This abnormal result triggered the investigation.This figure (diagram) shows that the error wasn’t caused by one mistake—but by multiple system failures in sequence in the presence of failures to detect the error.

Navigate back to Figure 3..
